# Excessive intake of iodine and low prevalence of goiter in school age children five years after implementation of national salt iodization in Shebedino woreda, southern Ethiopia

**DOI:** 10.1186/s12889-021-10215-y

**Published:** 2021-01-19

**Authors:** Elilta Elias, Workneh Tsegaye, Barbara J. Stoecker, Tafere Gebreegziabher

**Affiliations:** 1 School of Nutrition, Food Science and Technology, Hawassa University, P.O. Box 5, SNNPR, Hawassa, Ethiopia; 2 Department of Nutritional Sciences, Oklahoma State University, 421 Human Sciences, Stillwater, OK 74078 USA; 3 Department of Health Sciences, Central Washington University, 400 E University way, Ellensburg, WA 98926 USA

**Keywords:** Excessive iodine, Iodization, Goiter, School children, Southern Ethiopia

## Abstract

**Background:**

Iodine is a trace element required for the synthesis of thyroid hormones. The multiple effects of iodine deficiency on human health are called iodine deficiency disorders (IDDs). IDDs have been common nutritional problems in Ethiopia. In 2012, Ethiopia launched a national salt iodization program to address IDDs. The objective of this study was to assess the effects of this program after 5 years by measuring urinary iodine concentration (UIC) and prevalence of goiter in school age children as well as household salt iodine concentration (SIC).

**Methods:**

A school-based cross-sectional design was employed. After ethical approval, 408 children from eight randomly selected primary schools provided urine samples. UIC was analyzed by inductively coupled plasma mass spectrophotometry (ICP-MS). A 10 g salt sample was collected from each household of a sampled child. SIC was analyzed with a digital electronic iodine checker (WYD, UNICEF) and goiter was assessed by palpation.

**Results:**

The mean (±SD) age of the children was 9 ± 2 years. The prevalence of goiter was 4.2% and no child had grade 2 goiter. The median (IQR) UIC was 518 (327, 704) μg/L and UIC ranged from 3.1 to 2530 μg/L. Of the salt samples, 15.6% were not adequately iodized (< 15 ppm), 39.3% were adequately iodized (≥15 to ≤40 ppm), and 45.1% were > 40 ppm. SIC ranged from 4.2 to 195 ppm. Of the mothers, 92% said iodized salt prevents goiter and 8% mentioned prevents mental retardation.

**Conclusions:**

In 2017 iodine deficiency was no longer a public health problem in the study area. However, the high variability in UIC and SIC and excessive iodine intake are of great concern. It is vital to ensure that salt is homogenously iodized at the production site before being distributed to consumers.

## Background

Iodine is an essential trace element for human growth and development and is required for the synthesis of thyroid hormones [1]. Low levels of thyroid hormones result in hypothyroidism which could cause serious functional and developmental disorders collectively called iodine deficiency disorders (IDDs) [2]. These can have immediate negative effects on children’s learning capacity and school performance. If the trend continues, IDDs can lead to lower economic productivity and poor quality of life [2].

Currently, the preferred way to alleviate iodine deficiency is to increase iodine intake through implementation of universal salt iodization (USI). USI is the iodization of all salt in the country for human and livestock consumption [3]. However, despite national and international efforts to increase iodine intake, primarily through salt iodization programs, about 35% of the world’s population and nearly 30% of school-age children (241 million) were estimated in 2014 to have insufficient iodine intake. The majority (134 million) of these lived in South-East Asia and Africa [4, 5]. In 2017, iodine deficiency was still considered a global public health problem [6].

Although USI is the best strategy to alleviate IDDs, it requires a close monitoring system and may take time until there are changes in the prevalence of signs of iodine deficiency, particularly goiter. According to the Ethiopian Public Health Institute, in 2015, 3 years after launch of mandatory iodization of salt for humans, 47.5% of Ethiopian children had low UIC. The problem was worse in the southern parts of the nation where the percentage of school age children with urinary iodine of less than 100 μg/L was as high as 56.7%, and iodine deficiency remained a severe public health problem [7]. However, 5 years after the iodization program was launched, 29% of school children in North-West Ethiopia had excessive iodine intake, 62% had adequate iodine intake and the median urinary iodine concentration (UIC) was 235 μg/L. Prevalence of goiter was a severe public health problem as 34% of the school children had palpable or visible goiter [8].

Although there are several studies from Ethiopia before the salt iodization program, there is a lack of information about iodine status of school children following the iodization program, specifically those in the study area. This study was designed to assess iodine status and prevalence of goiter among school age (6–12 year old) children in Shebedino *woreda,* Sidama Zone, Southern Ethiopia 5 years after the national salt iodization program.

## Methods and materials

### Study design

This study employed a school-based cross-sectional design. Data were collected from May 10 to June 11, 2017.

### Description of study area and study population

The study was conducted in Shebedino *woreda (district)*, Sidama Zone, Southern Nations, Nationalities, and Peoples Region (SNNPR). Ninety-five percent of the population depend on mixed farming, 3.8% depend on trade and only 1.2% work for either governmental or non-governmental organizations (NGOs). All students in randomly selected primary schools of Shebedino *woreda* were the study population.

### Inclusion criteria

All children (6–12 years old) whose parents/guardians gave consent for their children to be part of the study were eligible to be included. No child included in the study was more than 12 years old.

### Exclusion criteria

Based on the exclusion criteria, children to be excluded were those who had attended the targeted schools for less than 6 months prior to the study and those who were chronically or acutely ill at the time of data collection. Because there were no students in these categories, no students were excluded from the study.

### Sample size

A single proportion sample size calculation formula was used to determine optimal number for estimating the prevalence of iodine deficiency. A sample size of 408 school age children was computed based on an estimated 59.1% prevalence of goiter [9], a 95% confidence level, 5% margin of error, design effect of 1.5% and non-response rate of 10%.

### Sampling technique

The woreda has 37 primary schools, out of which eight schools were selected by simple random sampling. The sample was allocated for each school by population proportional to size. The sampling frame was prepared by using registers that listed children 6–12 years of age in all selected schools. Finally, systematic random sampling was used to select the study participants from each school.

### Data collection method

Mothers were interviewed individually in their homes using a pretested structured questionnaire. Questions about socio-demographic and economic data were adapted from the Ethiopia Demographic and Health Survey (EDHS) questionnaire [10], and food consumption patterns were assessed using a standardized food frequency questionnaire [11]. The questionnaire was prepared in English, translated into the local language (Sidamigna) and translated back into English before data collection to check for consistency and to keep its contextual equivalence. Data were collected by four trained health professionals fluent in the local language. A 10 g salt sample was collected in a tightly sealed plastic container from each household of a sampled child during interview with the mother. The plastic containers were labeled with school code and children’s identification number. The salt samples were stored in a dry place until analyzed for iodine concentration.

Each child provided a urine sample at school in disposable plastic cups, and urine was transferred to screw-capped plastic vials. The vials were labeled with the school code and children’s identification numbers using both permanent marker and pencil. Plastic vials were sealed with Parafilm® to prevent leakage and evaporation. The urine samples were immediately placed in a thermo cool box and were transported to Hawassa University and stored at -20 °C until shipped frozen to the USA for analysis by ICP-MS.

### Examination for goiter

Children were examined for goiter at school by a single public health officer experienced in palpation. Stage of goiter was based on WHO criteria: grade 0, no palpable or visible goiter; grade 1, palpable goiter but not visible when neck is in the normal position; grade 2, visible goiter when neck is in the normal position [2].

### Laboratory analysis

Urinary iodine concentration was analyzed by inductively coupled plasma mass spectrometry (ICP-MS, Elan 9000, Perkin Elmer, Norwalk, CT) at Oklahoma State University, USA. Urine was diluted in 2% ammonium hydroxide and iodine was analyzed with tellurium as internal standard (Caldwell et al., 2003). Quality control samples for urinary iodine were measured every 10 samples to ensure stability of the instrument.

Salt iodine concentration (SIC) was analyzed with a digital electronic iodine checker (WYD, UNICEF) at Hawassa University, Ethiopia. All procedures were performed according to the instructions provided in the WYD manual. From a standard KIO_3_ solution containing 1000 μg/mL of iodine, 5 ml was diluted to 500 mL to make a 10 μg/mL working standard solution. A calibration solution was then made by combining 5 mL of the working standard solution, 2 mL of KI-starch solution, 2 mL (1 mol/L) sulfuric acid and further diluting with distilled water to a total of 50 mL and shaking well. After calibrating a zero reading on the WYD instrument using distilled water, the instrument’s liquid crystal display (LCD) reading was calibrated to 50.0 ± 0.1 using the calibration solution. For sample measurement, 1 g of well-mixed salt was dissolved in 10 mL of distilled water and the same quantities of KI-starch and sulfuric acid solutions were added with additional distilled water to make a total volume of 50 mL and mixed by shaking well. Salt solution samples were individually placed in the cell of the WYD iodine checker and iodine concentration was read on the LCD screen.

### Statistical analysis

Data were analyzed using SPSS, version 23 (SPSS Statistics Version 23, IBM Corp., Armonk, NY, USA). Descriptive analysis was used to report characteristics of the study participants.

Mean (SD) and median (IQR) were employed for descriptive statistics. Normality for distribution of continuous data was checked using a histogram with line curve for a visual test as well as the one sample Kolmogorov-Smirnov test. Natural log transformation was used to transform positively skewed data. Independent-sample t-test was used to compare means of UIC between male and female children.

## Results

A total of 408 mother-child dyads participated in this study. The mean age (±SD) of the mothers was 35 ± 8 years and of the children was 9 ± 2 years (Table 1). Most of the women had no formal education.

**Table 1:** Socio-demographic characteristics of school age children and their mothers in Shebedino woreda, southern Ethiopia, 2017 (*n* = 408)

Variables	Percent
Sex of child	
- Female	50.5
- Male	49.5
Age of child (years)	
- 6–8	42.2
- 9–12	57.8
Family size	
- 2–4	21.3
- 5–7	62.9
- ≥ 8	15.8
Age of mothers (years)	
- 21–28	15.7
- 29–35	49.0
- 36–44	21.6
- ≥ 45	13.7
Education of mothers	
- No formal education	52.5
- Able to read and write	2.5
- Primary school	40.6
- Middle or high school	4.4

As indicated in Table 2 most of the children consumed corn and enset at least once a day, followed in frequency by kale and haricot bean. More than 95% of the children had never consumed sweet potato, cassava, tomato, papaya, meat, chicken, egg, fish or cheese in the last month. A majority of the children consumed potato or avocado at least once a month. Milk, one of the potential good sources of dietary iodine, was consumed more frequently.

**Table 2:** Food consumption patterns of school age children in Shebedino woreda, Southern Ethiopia, 2017 (*n* = 408)

Food types	Frequency (%)			
	At least once/day	3-6x/week	At least once/month	Never
Cereals				
- Teff	0	3 (0.7)	23 (5.7)	382 (93.6)
- Wheat	4 (1)	3 (0.7)	39 (9.6)	362 (88.7)
- Corn	387 (94.9)	14 (3.4)	2 (0.5)	5 (1.2)
Legumes				
- Haricot bean	107 (26.2)	95 (23.2)	176 (43.2)	30 (7.4)
- Bean	4 (1)	26 (6.4)	77 (18.9)	301 (73.7)
Root crops & vegetables				
- Potato	11 (2.7)	71 (17.4)	242 (59.3)	84 (20.5)
- Sweet potato	0	3 (0.7)	13 (3.1)	392 (96.1)
- Cassava	3 (0.7)	3 (0.7)	14 (3.4)	388 (95.1)
- Enset	352 (86.3)	46 (11.2)	7 (1.7)	3 (0.7)
- Tomato	1 (0.2)	2 (0.4)	16 (3.9)	389 (95.3)
- Kale	202 (49.5)	184 (45.1)	18 (4.4)	4 (1)
Fruits				
- Avocado	12 (3)	34 (8.3)	270 (66.2)	92 (22.5)
- Papaya	0	0	16 (3.9)	392 (96)
- Banana	6 (1.4)	15 (3.7)	161 (39.5)	225 (55.1)
Meat, egg or fish				
- Meat (beef, sheep or goat)	0	0	16 (3.9)	392 (96.1)
- Chicken	0	0	5 (1.2)	403 (98.8)
- Egg	0	1 (0.2)	11 (2.7)	396 (97.1)
- Fish	0	0	3 (0.7)	405 (99.3)
Dairy products				
- Milk	14 (3.4)	17 (4.2)	239 (58.6)	138 (33.8)
- Cheese	3 (0.7)	0	10 (2.4)	395 (96.8)
- Butter-milk	24 (5.9)	48 (11.7)	259 (63.5)	77 (18.9)

The majority of the mothers said they had heard about iodine, iodized salt or IDD before and their primary source of information was health workers. More than 90% of the respondents said they knew about the benefits of iodized salt; however 92% of those said it prevents goiter but only 8% related lack of iodine to mental retardation (Table 3).

**Table 3:** Knowledge of mothers about iodized salt and IDD in Shebedino woreda, southern Ethiopia, 2017 (*n* = 408)

	Percent
Heard about iodine or IDD	
- Yes	75.2
- No	24.8
Heard about iodized salt	
- Yes	91.7
- No	8.3
Source of information	
- Health worker	60
- Mass media	27.2
- Public announcement	12.8
Know benefit of iodized salt	
- Yes	90.7
- No	9.3
Benefits of iodized salt	
- Prevents goiter	92
- Prevents mental retardation	8

**Fig. 1:**
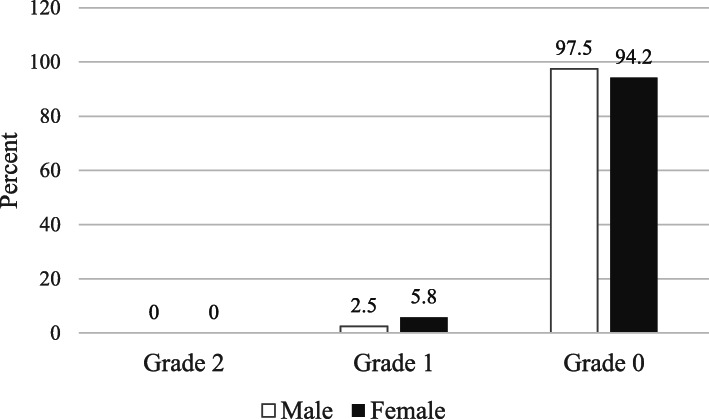
Prevalence of goiter in school age children in Shebedino woreda, southern Ethiopia, 2017 (*n* = 408)

**Fig. 2:**
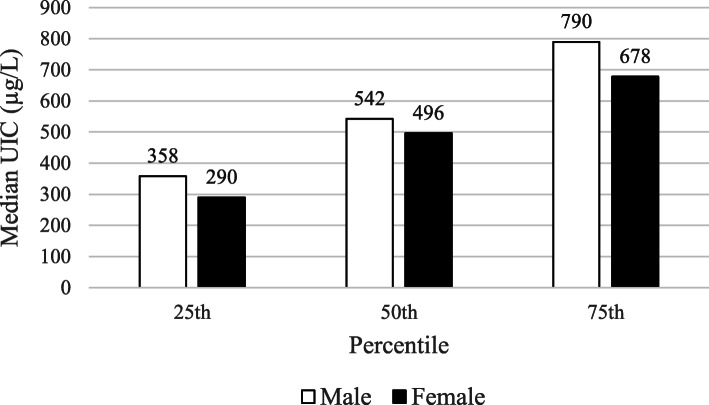
Median UIC of school age children in Shebedino woreda, southern Ethiopia, 2017 (*n* = 408)

As shown in Fig. 1, prevalence of goiter in school children was very low. Only 12 female and 5 male children had grade 1 goiter; the rest did not have goiter. This was consistent with their UIC. The median (IQR) for UIC of children was 518 (327, 704) μg/L. Mean (SD) UIC of male children was 581 (311) μg/L and female children was 518 (310) μg/L and this was significantly different (*p* = 0.043). Fewer than 5% of children had UIC less than 100 μg/L. More concerning was that even the 25th percentile for UIC was greater than 300 μg/L (Fig. 2).

**Fig. 3:**
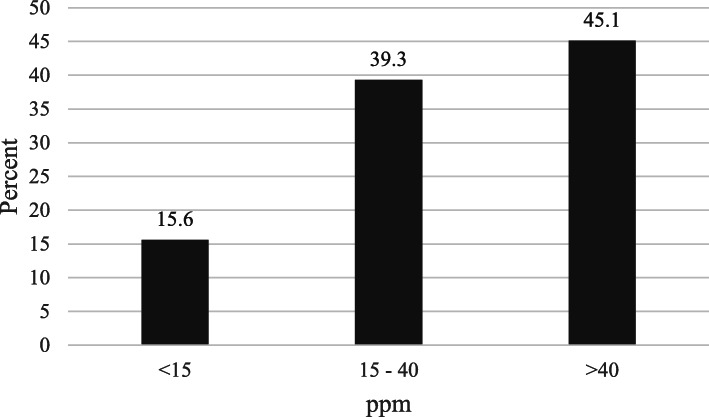
Household salt iodine concentration in Shebedino woreda, southern Ethiopia, 2017 (*n* = 408)

Most salt from households contained ≥15 ppm iodine. Among the 408 samples collected from each household, none were non-iodized. Of the collected samples 15.6% were inadequately iodized (< 15 ppm), 39.3% were adequately iodized (15 ppm – 40 ppm) and 45.1% had more than 40 ppm iodine (Fig. 3). The median (IQR) iodine concentration of the salt was 37 (22, 55) ppm and the range was 4.2 to 195 ppm.

## Discussion

Iodine deficiency had been a serious problem throughout Ethiopia, including the study region, for many years. Repeated studies conducted before the launch of the national salt iodization program in the country, particularly in SNNPR, showed that iodine deficiency was a serious public health problem [12–15]. In order to alleviate this long-standing problem, the national salt iodization program was launched in 2012 and iodized salt became available as a source of iodine in the study region [15, 16].

The current study showed high urinary iodine concentration (UIC) and low prevalence of goiter following national salt iodization. Prevalence of goiter in school age children in the study region in 2007 was 56.2% [12], and in the current study it was only 4.2% which indicates successful elimination of iodine deficiency as a major public health problem according to WHO/UNICEF/ICCIDD criteria [2]. Similarly, in a nine year prospective study in Iran, total goiter rate reportedly decreased from 44% in 1996 to 7.6% in 2001 and to 0.4% in 2007 following their national salt iodization program [17].

According to the WHO/UNICEF/ICCIDD recommended criteria, a population without iodine deficiency should have median UIC between 100 μg/L and 299 μg/L and UIC should not be lower than 50 μg/L in more than 20% of subjects [18]. In the current study the median UIC was 518 μg/L; more than 95% of the children had UIC above 100 μg/L and only 4.3% had UIC < 100 μg/L indicating that iodine deficiency has been eliminated for the population. However, 78% of the children had UIC > 300 μg/L which reflects a risk for excessive iodine intake and could be a cause for adverse health consequences including iodine-induced hyperthyroidism and auto-immune thyroid diseases [19]. Because ours was a cross-sectional study, we do not know how long the children had been consuming household salt iodized at > 40 ppm. Risk of adverse effects of excessive iodine intake is expected 5 to 10 years following introduction of iodized salt, and the consequences of excessive iodine intake are worse in places where iodine deficiency previously existed [2, 20, 21]. Risk of excessive iodine intake and possible adverse effects have been reported in Ghana following their universal salt iodization program [22], and a study in China reported thyroid nodules in response to excess iodine intake [23].

The large variation in UIC observed in this study is of great concern. UIC ranged from 3.1 μg/L to 2530 μg/L. This could be due to the large variation observed in SIC, which varied from 4.2 to 195 ppm (mg/kg). Because salt had been iodized using knapsack sprayers [16], it is unlikely to be iodized homogenously which could be a reason for high variability in amounts of iodine in salt. Although not as high as the current study, school age children in northwest Ethiopia in 2016 had median UIC of 235 (161, 320) μg/L and 29.1% of the children had UIC that suggested excessive iodine intake [8]. In an urban area of central Italy, high variation of UIC (33.2–819.5 μg/L) was observed in children who used iodized salt [24]. Due to the potential health risks, iodine intake that produces UIC > 300 μg/L per day should be discouraged [2, 25]. Generally in the current study more than 45% of the salt samples were above 40 ppm, an indication of more than adequate iodization [2].

The reduced prevalence of iodine deficiency in the study area is promising. However, iodine deficiency was still a public health problem in some parts of the country [26–28]. A study conducted in the Robe district in 2015 showed that 57% of the participants had low UIC and the median UIC was 78 μg/L [29]. In another study, nearly 65% of the households in Southern Wollo reported inadequate use of iodized salt 6 years after the launch of the national salt iodization program [30]. Additionally adequate iodine intake in school-aged children doesn’t assure adequate intake in at risk populations even in a country with a salt iodization program. In Sweden for instance, school age children had adequate iodine intake while pregnant women had inadequate intakes [31].

The ultimate goal of a universal salt iodization program is to alleviate iodine deficiency. However, the process must be closely monitored to prevent both inadequate and excessive iodine intakes [32]. The marked regional variation in Ethiopia implies the need to study factors causing such disparities in availability of iodized salt at the household level.

The WHO recommends that 90% of households in a population should be able to obtain iodized salt with an iodine level > 15 ppm for the effective elimination of IDD [2]. In the current study, 84.3% of households used adequate or more than adequately iodized salt. Although the percentage of households is lower than the recommendation and despite the high variability, the use of iodized salt is a great achievement compared to the iodized salt coverage of only 3 to 5% of households shortly before implementation of the national program [33]. The concern is, however, that unless salt iodization programs are practiced as industry norms, government- funded projects are not sustainable. For example, in Vietnam a government-funded universal salt iodization program was not sustainable as household iodized salt coverage declined from 90% in 2005 to 45% in 2011 [34]. In Tunisia, only 50% of households used adequately iodized salt after two decades of salt iodization which shows the program failed to meet the goals of USI, that is > 90% of households should use adequately iodized salt [35].

Maternal knowledge and practices are key factors for addressing public health problems. Low level of knowledge was associated with increased prevalence of IDD and has been identified as a major constraint in eliminating IDD in some African countries [36, 37]. In the current study, most of the women had heard about IDD, iodine and iodized salt. This is a remarkable improvement compared to previously reported studies in the region [13, 38]. Handling and storage conditions of iodized salt could also affect its iodine content. According to WHO, 20% of iodine from salt may be lost between production and the household, and another 20% of the iodine could be lost during cooking [2, 38]. However, in this study more than 97% of the respondents said they stored their salt in a dry place with a covered container which helps retain iodine in salt.

Because schools and children were randomly selected for this study, we are confident that our sample was representative of the study community and local region. However, Ethiopia is a large country with many agroecological zones. Because soil types and water sources contribute to diets of subsistence farming households [39], a study in a single region cannot be nationally representative. Furthermore, the current study did not analyze iodine concentration of consumed foods which was a limitation.

## Conclusions

In conclusion, although prevalence of goiter was low, based on the median UIC the school children could be assumed to be at risk of excessive iodine consumption. The salt iodization process needs to be monitored strictly before the population suffers detrimental health effects from excessive intake. Nonetheless, since this analysis was done on spot UIC samples, repeated measures at different times and in different population groups are warranted.

## Data Availability

The dataset used and/or analyzed during the current study are available from the corresponding author on reasonable request.

## Abbreviations

UIC: Urinary iodine concentration
SIC: Salt iodine concentration
IDDs: Iodine deficiency disorders
ICP-MS: Inductively coupled plasma mass spectrometry
IQR: Interquartile range
USI: Universal salt iodization
WHO: World Health Organization
SNNPR: Southern Nations, Nationalities and Peoples Region
NGO’s: Non-governmental organizations
EDHS: Ethiopia Demographic and Health Survey
